# Fetal alcohol and the right to be born healthy…

**DOI:** 10.3389/fgene.2014.00356

**Published:** 2014-10-13

**Authors:** Shiva M. Singh, Benjamin I. Laufer, Joachim Kapalanga

**Affiliations:** ^1^Molecular Genetics Unit, Department of Biology, The University of Western OntarioLondon, ON, Canada; ^2^Department of Neuroscience, The University of Western OntarioLondon, ON, Canada; ^3^Department of Pediatrics, Schulich School of Medicine and Dentistry, The University of Western OntarioLondon, ON, Canada

**Keywords:** teratogen, spectrum disorders, neurodevelopment, neuroepigenomics, pregnancy, exposure, environment, fetal alcohol syndrome

Finding the cause and applying the insights toward prevention and treatment forms the ultimate goal of most disease research. This strategy has been successfully used to make diseases like Scurvy and Smallpox, a history. The impact of this research during the last few years has been nothing less than miraculous. More and more people are living longer with healthy and productive lives, well into the 80's and 90's. Discovery of the cause(s) of disease(s) however is a demanding, time consuming, and expensive exercise. Also, there is no guarantee for success. Yet, even the modest success in search for causes have the potential to change the outcome and perception. Early diagnosis of a number of cancers for example is now viewed as treatable with reasonable chance of recovery. Also, some heart diseases are being managed and treated with high rate of success. Given this record of success, the research on disease causations continues to increase and the results have begun to pay increasing dividend. Unfortunately, there are cases of diseases where even full understanding of the cause has not resulted in the prevention or treatment of some common and devastating diseases. One such disease is the fetal alcohol spectrum disorder (FASD).

FASD is caused by the exposure of developing fetus to alcohol via maternal drinking during pregnancy (Jones and Smith, 1973). It represents the biggest single cause of mental retardation and developmental disabilities among babies born in the Western World (Barry et al., 2009). In the U.S. more than 50,000 babies are born with FASD every year (May and Gossage, 2001) and the annual cost of treating FASD in Canada and U.S. exceeds $6 and $8 billions, respectively (Lupton et al., 2004; Popova et al., 2013). Although, the prevention of FASD is a high priority, the failure to prevent it is attributed to our alcohol culture. Most people drink for social and recreational purposes. Others are addicted to alcohol.

As it stands, there is no consensus on whether there is a “safe” limit for alcohol consumptions during pregnancy. Recent research involving animal (mice) models has shown that continuous exposure of low-to-moderate dose of alcohol during pregnancy impacts behavioral and cognitive outcomes of resulting pups (Kleiber et al., 2011) and even a single binge dose of alcohol at any time during pregnancy results in alterations in gene expression (Kleiber et al., 2012, 2013) and associated FASD related phenotypes. Furthermore, the molecular alterations may be initiated and maintained for life by alcohol's effect on epigenetic features that includes DNA methylation (Laufer et al., 2013). The results on animal models argue that clinical features of FASD represent “tip of the iceberg.” They are also backed by results on humans. For example, exposure of human embryonic stem cells to low alcohol can alter gene expression leading to the abnormal development of prefrontal cortex (Krishnamoorthy et al., 2010). Also, fetal alcohol exposed school children show “a small but potentially important detrimental effect” on educational outcomes (Zuccolo et al., 2013) as well as generalized deficit of conceptualization (Quattlebaum and O'Connor, 2013).

We feel that such results deserve due consideration given that Royal College of Obstetrics and Gynecologists (Royal College of Obstetrician and Gynaecology, 2006) states that, “there is no evidence of harm from low levels of alcohol consumption, defined as no more than one or two units of alcohol once or twice a week.” Also, “there is considerable doubt as to whether infrequent and low level of alcohol consumption during pregnancy convey any long-term harm”—in other words they suggest a safe amount of alcohol consumption in pregnancy. Unfortunately, this limit has not been defined and may vary from individual to individual. Individual women process alcohol differently. Also, the age of the mother, the timing and regularity of the alcohol ingestion, and whether the mother has eaten any food while drinking may be important. We argue that there is no logistic evidence to define this limit. What is needed is to undertake thorough studies on neurodevelopment and assess the significance of such factors as maternal and fetal genotype, stress during pregnancy and childbirth, prenatal drinking patterns (mild, medium, heavy), post-natal environment, and socioeconomic status, as most of these may contribute to the manifestation of the effect of prenatal alcohol on the newborn. We note that some of these studies will be problematic if not impossible on humans. The rational question is “does no evidence of harm from low levels of alcohol consumption means 100% exclusion of the possibility of any harm to the fetus?” To the best of our understanding the answer is “no.”

The issue is particularly problematic as there is a rise in heavy drinking by young people, particularly women. Often, it is framed, as freedom of choice or “a single drink will not harm.” Not surprisingly, 1 in 8 adult women and 1 in 5 high school girls binge drink (CDC, 2013) and there is ample evidence from animal experiments, which argue for a life-long effect of even a single exposure of alcohol during pregnancy. The developing brain is a sequential, multistage, closely orchestrated, and highly sensitive to stresses. Also, any aberration could lead to life-long abnormality. For now, it is prudent to prevent a brain disorder than to attempt to ameliorate or cure it. Preventing a single case of FASD will save the society $1 million. More importantly it will save a productive life. The business as usual model is not helpful. It continues to result in births with alcohol effects. Any harm caused by prenatal alcohol is currently not reversible. It will affect the child for life.

With the current knowledge of what causes FASD it is prudent to stay on the safe side and avoid any drinking during and around the pregnancy. FASD is an alcohol problem. It is possible to prevent this calamity by avoiding alcohol during pregnancy and the time is now! FASD is a preventable disease—by not drinking during pregnancy. On the other hand, finding “cure” will be much more challenging, costly and time taking. It is critical to undertake active measures to reduce the occurrence of this disorder by a message of “no alcohol dose is guaranteed to be 100% safe for the embryo/fetus.” Also, “no time during pregnancy is 100% safe to drink.” Any adult has the right to drink if they so wish. Also, every child has the right to be born healthy!

## Conflict of interest statement

The authors declare that the research was conducted in the absence of any commercial or financial relationships that could be construed as a potential conflict of interest.
